# Editorial: Endothelial-to-mesenchymal transition in cardiovascular disease

**DOI:** 10.3389/fcvm.2023.1290050

**Published:** 2023-10-12

**Authors:** Mabruka Alfaidi, Paul C. Evans, J. Geoffrey Pickering

**Affiliations:** ^1^ Department of Internal Medicine, Division of Cardiology, Louisiana State University Health Sciences Center at Shreveport, Shreveport, LS, United States; ^2^ Biochemical Pharmacology, William Harvey Research Institute, Barts and the London Faculty of Medicine and Dentistry, Queen Mary University of London, London, United Kingdom; ^3^ Departments of Medicine, Biochemistry, and Medical Biophysics, Western University, London, ON, Canada

**Keywords:** EndMT, cardiovascular diseases, pathogenesis, molecular pathways, endothelial-to-mesenchymal transition

**Editorial on the Research Topic** Endothelial-to-mesenchymal transition in cardiovascular disease

Endothelial-to-mesenchymal transition (EndMT) is a process through which endothelial cells (ECs) transition into mesenchymal cells and gain invasive and migratory properties. During this process, ECs can delaminate from their cell layer and invade the underlying tissue. In the classical form of EndMT, this is accompanied by downregulation of EC markers such as CD31 and VE-cadherin with concomitant upregulation of mesenchymal markers such as α-SMA (alpha-smooth muscle actin) and PDGFRα (platelet-derived growth factor receptor alpha), vimentin (VIM), and N-cadherin (CDH2) (1). However, it is now known that EndMT can be partial (2) and in some cases transient (3). EndMT is a fundamental process during early development (4) and has also been identified in a multitude of cardiovascular disease processes, including atherosclerosis (5–8), valvular heart disease, peripheral artery disease (9), and myocardial infarction (10). Growing evidence for EndMT in human pathologies point to the clinical relevance of EndMT in cardiovascular diseases (5, 11, 12).

The collection of the research articles presented herein provides new insights into the molecular mechanisms and importance of EndMT in cardiovascular physiology and disease. The research highlights the complexities of the EndMT process, including distinctions among different EndMT-inducing stimuli and between heart valves and the vessel wall (Table 1). The relevant actions of TGFβ1, TGFβ2, TNFα, and flow, among other mediators, are explored.

**Table 1:** Summary to the endMT in cardiac valves vs. in vasculature and differences among species.

Authors	Study type	Species	Main findings
Andueza et al. (8)	Vascular wall remodeling in PCL model of disturbed flow	Mouse	- ScRNA and ScATAC-seq showed DF-promotes EndMT with upregulated Acta2, Snai1, Tagln, Cnn1.
Chen et al. (7)	Human coronary atherosclerosis samples Mouse HFD-induced atherosclerosis model Cell culture: HUVECs with fluid shear stress	Human HUVECs Mouse	- Immunofluorescence staining of human coronary atherosclerosis showed co-localization between FN/ICAM-1, CD31/FN, CD31/NOTCH3, CD31/SM22α, P-SMAD2/FGFR1. - OSS downregulated FGFR1 and upregulated nuclear SMAD2/3 translocation. OSS increased EndMT markers: TWIST1, ACTA2, NOTCH3, N-cadherin, FN, Collagen 1A. - Suppression of FGF signaling increased atherosclerosis, and EndMT (histological assessment of ICAM-1/FN/Chd5-GFP).
Chen et al. (13)	Cell culture: HUVECs treated with TGFβ1 HFD-induced atherosclerosis	HUVECs Mouse	- TGFβ1 stimulation induced markers of inflammation. ECM and MMPs were upregulated. - TGFR1 and 2 EC-specific KO mice showed less atheroma compared to controls. Some but not all EndMT clusters were abolished.
Evrard et al. (5)	HFD-induced atherosclerosis Human atherosclerotic aorta Cell culture	Mouse Human HUVECs	- Using Lineage tracing mice, ECs were found to give rise to Fap + cells (EndMT with fibroblast-like features). Fap + cells were increased in the cap with more chronic HFD feeding. - After 30 weeks, many Fap + cells did not express VE-cadherin. - Human atherosclerosis intimal cells co-expressed fibroblast and endothelial cell markers (FSP-1/CD31, FSP-1/VWF). - TGFβ alone did not induce the EndMT with fibroblasts-like features H_2_O_2_ or hypoxia were also required to push the phenotype. Cell migration was used as a functional assay.
Moonen et al. (18)	Ex vivo TAC-induced mouse model Cell culture	Human Pig aortic tissue Mouse HUVECs/HAECs	- Human endarterectomy carotid atherosclerosis samples showed cells double-positive for PECAM-1/αSMA - Porcine aorta showed cells double-positive for endocan/transgelin or endocan/αSMA, transgelin, and calponin in only areas of DF but not in LSS areas. - HUVECs and HAECs treated with TGFβ1 induced EndMT which was prevented by LSS.
Mahmoud et al. (19)	Ex vivo HFD-induced atherosclerosis Zebrafish model	Pig aortic tissue HUVECs Mouse/Zebrafish	- TWIST1 and GATA4 co-localized with CD31 were preferentially expressed in low shear areas in porcine aorta. - The orbital shaker model was used to assess TWIST1/CD31 and GATA4/CD31 - Positive cells in culture after 72 h. - Twist1 KO in ECs reduced atherosclerosis. - Zebrafish embryos: twist1 enhanced under static vs flow. - Proliferation was assessed as a functional assay for EndMT.
Zhang et al. ^a^	In vitro—Cell culture HFD-induced atherosclerosis	HAECs Mouse	- TGFβ2 promoted EndMT after 3 days in culture: downregulation of CD31/VE-cadherin, upregulation of α-SMA/PDGFRα. - The EndMT was reversed after removal of TGFβ2 by 2 days. - HFD in LDLR^−/−^ mice induced some of the phenotype.
Tombor et al. (3)	LAD-ligation model of MI	Mouse HUVECs	- scRNA seq of non-cardiomyocytes showed induction of mesenchymal cells with increase in FN1, Vimentin, Serpine1, MMP14. - Cdh5-lineage tracing confirmed induction of GFP + ECs with mesenchymal markers: Col1a1, Col3A1, Serpine 1. - TGFβ2 treatment in HUVECs after 3 days induced calponin, SM22, and withdrawal of TGFβ2 reversed the process.
Bischoff et al. (20)	Ovine MI model	Ovine Mitral valve ECs	- TGFβ1 for 96 h induced EndMT (αSMA/VE-cadherin) in ovine mitral valve not carotid ECs. - Cell migration was used to assess the functional changes.
Kim et al. (21)	Myxomatous valve disease model	Mouse	- EndMT (GFP+/αSMA) was not seen in adult murine valves when analyzed by *in vivo* EC-specific lineage tracing model.
Nehl et al. ^a^	Ex vivo	Porcine aortic valve Human aortic valve	- After 7 days of TGFβ1 stimulation of human valvular ECs, the endothelial marker VWF was downregulated, but PECAM-1 and VE-cadherin were upregulated. Stimulation with TNFα for 7 days resulted in downregulation of EC markers, including VWF, PECAM-1, NOS3, and upregulation of the mesenchymal markers α-SMA, VIM, CDH2, and VCAM-1. - Porcine valvular ECs did not show the same response. - Migration (scratch wound) and calcification were used for functional assay.
Zhong et al. (22)	Cell culture: cells treated with TGFβ1, TNF-α, or H_2_O_2_ Ex vivo: Porcine aortic valves	Porcine aortic valve ECs.	- TGFβ1 treatment for 6 days, but not TNF-α, nor H_2_O_2_ induced VECs to downregulate VE-cadherin and express α-SMA. - This phenotype was enhanced by matrix stiffness (50kPa). - Endostatin blocked the phenotype.

DF, disturbed flow; ECs, endothelial cells; ECM, extracellular matrix proteins; FN, fibronectin; FSP-1, (fibroblast-specific protein-1); HFD, high fat diet; HAECs, human aortic endothelial cells; HUVECs, human umbilical vein endothelial cells; ICAM-1, intercellular adhesion molecule-1; LSS, laminar shear stress; MMPs, matrix metalloproteases; OSS, oscillatory shear stress; PCL, partial carotid ligation.

^a^ Indicates studies highlighted in this issue.

Zhang et al. present a novel mechanistic link between TGFβ2 and Wnt signaling pathway in human aortic endothelial cells and mouse atherosclerotic plaques. Exposure of cultured endothelial cells to TGFβ2 for 3 days upregulated α-SMA and PDGFRα and downregulated CD31 and VE-cadherin. After removal of TGFβ2 from the media, endothelial cell adhesion marker genes re-expressed, highlighting the plasticity of the response. Interestingly, deletion of Wnt2 significantly abolished the TGFβ2- driven EndMT. Wnt2 also colocalized with α-SMA in aortic atherosclerosis in LDLR^−/−^ mice fed Western diet for 12 weeks, but not in the chow diet-fed mice, indicating that Wnt2 expression is associated with atherosclerosis. A recent study by Chen et al. (13) reported that TGFβ induced populations of EndMT with proinflammatory features, and that Wnt signaling was altered upon EC-specific knock-out of TGFβ receptors 1 and 2 in atherogenic mice. The interplay among TGFβ2-mediated EndMT formation and Wnt2 in regulating the atherosclerosis burden is thus a key topic for further study.

Another disease context for EndMT is calcified aortic valve disease (CAVD) (14). Valvular endothelial cells can undergo EndMT and transdifferentiate into myofibroblast-like cells, with subsequent immune cell infiltration and calcification (15, 16). There is no medical treatment currently for CAVD and delineating molecular and cellular mechanisms of EndMT in this disease thus merits attention (17). Nehl et al. isolated ECs from porcine and human valvular tissue and exposed the cells with TGFβ1 or TNFα. Interestingly, the phenotype responses differed considerably between stimuli. After 7 days of TGFβ1 stimulation of human valvular ECs, the endothelial marker VWF (Von Willebrand factor) was downregulated, but PECAM-1 and VE-cadherin were upregulated. In contrast, stimulation with TNFα for 7 days resulted in consistent downregulation of EC markers, including VWF, PECAM-1, NOS3, and upregulation of the mesenchymal markers α-SMA, VIM, CDH2, and VCAM-1. The porcine valvular ECs on the other hand did not substantially change their phenotype markers, nor was their migratory response like that of the human valvular ECs. This research highlights the diversity of EndMT profiles depending on the stimulus and potential for species-specific responses.

Also in this issue, Chen et al. review the emerging concept of EndMT as “an extreme spectrum of endothelial activation”. The authors discuss TGFβ as a major inducer of EndMT. Disturbed flow was also sufficient to induce EndMT and under disturbed flow, FGF (Fibroblast growth factor) was downregulated, which exerts a positive effect on TGFR1. FGF and TGFβ have reciprocal actions in this regard. Furthermore, gene expression data analyses of ECs vs. cells having undergone EndMT vs. fibroblasts suggest that the set of EndMT genes differs from both ECs and fibroblasts.

In another review, Huang et al. provide a discussion of the mechanisms of EndMT in atherosclerosis and the different stimuli used by researchers to induce EndMT in culture, including TGFβ, interleukin-1 (IL-1β), oxidized low-density lipoprotein (oxLDL), Hydrogen peroxide (H_2_O_2_), and shear stress. The TGFβ signaling pathway, bone morphogenic protein (BMP) signaling pathway and NOTCH signaling pathway in EndMT induction are reviewed. Preventing EndMT to treat atherosclerosis is considered Huang et al. Finally, Jiang et al. review the role of EndMT in vascular calcification, also with consideration to therapeutic strategies.

Collectively, these research articles and reviews add to our understanding of EndMT in cardiovascular disease. The diversity of phenotypes and the differences among drivers of EndMT highlight the complexity of this remarkable re-wiring of endothelial cells. Ultimately, proving disease-altering roles for EndMT requires further attention, with the exciting possibility of disease-mitigating strategies.
